# An avian live attenuated master backbone for potential use in epidemic and pandemic influenza vaccines

**DOI:** 10.1099/vir.0.2008/004143-0

**Published:** 2008-11

**Authors:** Danielle Hickman, Md Jaber Hossain, Haichen Song, Yonas Araya, Alicia Solórzano, Daniel R. Perez

**Affiliations:** Department of Veterinary Medicine, University of Maryland, College Park and Virginia-Maryland Regional College of Veterinary Medicine, 8075 Greenmead Drive, College Park, MD 20742-3711, USA

## Abstract

The unprecedented emergence in Asia of multiple avian influenza virus (AIV) subtypes with a broad host range poses a major challenge in the design of vaccination strategies that are both effective and available in a timely manner. The present study focused on the protective effects of a genetically modified AIV as a source for the preparation of vaccines for epidemic and pandemic influenza. It has previously been demonstrated that a live attenuated AIV based on the internal backbone of influenza A/Guinea fowl/Hong Kong/WF10/99 (H9N2), called WF10*att*, is effective at protecting poultry species against low- and high-pathogenicity influenza strains. More importantly, this live attenuated virus provided effective protection when administered *in ovo*. In order to characterize the WF10*att* backbone further for use in epidemic and pandemic influenza vaccines, this study evaluated its protective effects in mice. Intranasal inoculation of modified attenuated viruses in mice provided adequate protective immunity against homologous lethal challenges with both the wild-type influenza A/WSN/33 (H1N1) and A/Vietnam/1203/04 (H5N1) viruses. Adequate heterotypic immunity was also observed in mice vaccinated with modified attenuated viruses carrying H7N2 surface proteins. The results presented in this report suggest that the internal genes of a genetically modified AIV confer similar protection in a mouse model and thus could be used as a master donor strain for the generation of live attenuated vaccines for epidemic and pandemic influenza.

## INTRODUCTION

In the 20th century, humans experienced three pandemics of influenza with significant death tolls (Horimoto & Kawaoka, 2001). The emergence of highly pathogenic H5N1 avian influenza virus (AIV) in Asia, with an unusually broad host range and the ability to infect and kill people, has raised concerns that another pandemic is looming over us (Horimoto & Kawaoka, 2001). Vaccines are undoubtedly a major resource that can greatly reduce the impact of the pandemic. Currently, two types of vaccine are commercially available for the prevention of epidemic influenza in the USA: inactivated whole virion and live attenuated vaccines (Belshe, 2004; Harper *et al.*, 2004; Zangwill & Belshe, 2004). FluMist is a live attenuated influenza vaccine composed of the three dominant circulating strains of human influenza virus types A and B. This vaccine has been shown to be efficacious and safe for delivery in children and adults aged 5–49 and is not transmissible to susceptible contacts (Belshe, 2004; Harper *et al.*, 2004). The two type A influenza viruses present within the FluMist vaccine are reassortants containing the surface genes of the currently circulating H1N1 and H3N2 strains and six internal genes from the master donor virus influenza A/Ann Arbor/6/60 (H2N2) (MDV-A) with a cold-adapted (*ca*), temperature-sensitive (*ts*) and attenuated (*att*) phenotype (Maassab, 1967).

Murphy *et al.* (1982, 1997) and Subbarao *et al.* (1995) developed alternative approaches for the generation of live attenuated vaccines for humans using reassortants between avian and human influenza A viruses. The main concept behind these latter approaches was based on the host-range restriction shown by AIVs. Thus, viruses carrying genes derived from an AIV would be attenuated in humans, whereas the presence of the human haemagglutinin (HA) and neuraminidase (NA) surface proteins would elicit a protective immune response against circulating influenza A viruses. These experimental vaccines showed great promise in pre-clinical studies and in clinical studies in adults and older children (Sears *et al.*, 1988; Steinhoff *et al.*, 1990). Unfortunately, some of these vaccines caused reactions in young children and infants, resulting in high fever and other flu-like symptoms. In addition, the consistent failure to obtain some of the reassortant viruses made these approaches impractical (Steinhoff *et al.*, 1990, 1991).

The advent of reverse genetics has opened up new alternatives for the development of live attenuated vaccines (Neumann & Kawaoka, 2001). This is particularly important considering the unprecedented emergence of multiple strains of AIVs with unexpectedly broad host ranges (Capua & Marangon, 2004). If one of these strains was spread among a broad range of animal species, we should expect major health, economic and ecological consequences. It is unrealistic to consider the preparation of multiple vaccine formulations specifically tailored for multiple animal species if such a strain were to emerge (Capua & Alexander, 2002, 2004; Capua & Marangon, 2004). We have previously analysed an AIV backbone that has shown a broad host range, influenza A/Guinea fowl/Hong Kong/WF10/99 (H9N2) (WF10), for its potential as a suitable virus vaccine donor that could be used in multiple animal species, including humans (Song *et al.*, 2007). H9N2 viruses of the same lineage as the WF10 virus have been shown to effectively infect multiple domestic poultry species, including ducks, turkeys, chickens and quail, as well as mice, without prior adaptation (Choi *et al.*, 2004; Guan *et al.*, 2000; Lin *et al.*, 2000; Peiris *et al.*, 1999, 2001; Perez *et al.*, 2003a, b; Xu *et al.*, 2004). Viruses phylogenetically related to the WF10 virus have also been isolated from pigs (Xu *et al.*, 2004). Furthermore, we have shown that the WF10 virus has many biological features similar to human influenza viruses, including the ability to infect non-ciliated cells in cultures of human airway epithelial cells (Wan & Perez, 2007). Thus, WF10 represents a potentially ideal candidate for the preparation of live vaccines applicable to multiple animal species. In previous studies, we showed that introduction of the MDV-A mutations into the WF10 virus in combination with an epitope HA tag in frame with the C terminus of the PB1 polymerase subunit resulted in a virus with an *att* phenotype and provided excellent protection in birds (Song *et al.*, 2007). In this study, we wanted to expand on the characterization of the genetically modified WF10 backbone as a vaccine donor for influenza viruses of other animal species. Our results showed that genetically modified WF10 reassortant viruses induced protective immunity against the highly lethal influenza A/WSN/33 (H1N1) and A/Vietnam/1203/04 (H5N1) strains in mice. Furthermore, vaccination with a heterologous subtype also resulted in adequate cross-protection. These studies highlight the potential of a genetically modified AIV backbone as a donor for influenza vaccines for avian and mammalian species.

## METHODS

### Cells and viruses.

293T human embryonic kidney and Madin–Darby canine kidney (MDCK) cells were maintained as described by Song *et al.* (2007). The WF10 and the mouse-adapted influenza A/WSN/33 (H1N1) (WSN) viruses were kindly provided by Dr Robert Webster, St Jude Children's Research Hospital, Memphis, TN, USA. The highly pathogenic AIV A/Vietnam/1203/04 (H5N1) (HPAI H5N1) was obtained from the repository at the Centers for Disease Control and Prevention, Atlanta, GA, USA. The A/Chicken/Delaware/VIVA/04 (H7N2) virus was kindly provided by Dr Dennis Senne, National Veterinary Services Laboratory, APHIS-USDA, Ames, IA, USA. The titre of stock viruses was measured by plaque assay on MDCK cells at 37 or 32 °C or by 50 % egg infectious dose (EID_50_) as described previously (Reed & Muench, 1938). All *in vitro* studies using HPAI virus were performed in an enhanced Biosafety Level 3 (BSL-3) facility approved by the US Department of Agriculture (USDA).

### Generation of recombinant viruses by reverse genetics.

The H7 and N2 genes of the H7N2 virus and ΔH5 (deletion of polybasic amino acids) and N1 genes of the HPAI H5N1 virus were cloned as described by Song *et al.* (2007). Recombinant viruses were generated by DNA transfection into co-cultured 293T and MDCK cells as described previously (Hoffmann *et al.*, 2000). Recombinant virus stocks were prepared in the allantoic cavity of 10-day-old embryonated chicken eggs. For each virus prepared, RT-PCR and sequencing were performed for each viral segment to determine their identity. Sequences were generated using a specific set of primers, a Big Dye Terminator v3.1 Cycle Sequencing kit (Applied Biosystems) and a 3100 Genetic Analyzer (Applied Biosystems), according to the manufacturer's instructions.

### Animal studies.

Five-week-old female BALB/c mice (Charles River Laboratories) were anaesthetized with isofluorane before intranasal inoculation with 50 μl virus suspension. The 50 % mouse lethal dose (MLD_50_) for the WSN, A/VN/1203/04 and recombinant viruses was calculated using groups of four mice inoculated intranasally (i.n.) with various doses ranging from 10^0^ to 10^6^ p.f.u. per mouse so that mice could be challenged in later experiments with 20 MLD_50_. Clinical symptoms, body weight and mortality of mice were monitored and recorded for 14 or 21 days as indicated. Animal studies using H1N1 recombinant viruses were conducted under BSL-2 conditions, whereas those with H5N1 (HPAI) recombinants were performed under BSL-3 conditions with USDA approval. Animal studies were performed according to protocols approved by the Animal Care and Use Committee of the University of Maryland.

### Evaluation of the protective efficacy of recombinant viruses.

To evaluate the induction of immune responses and the protective capacity of the recombinant viruses against wild-type WSN virus challenge, mice (seven per group) were immunized i.n. with recombinant viruses in a 50 μl volume at various doses ranging from 10^3^ to 10^6^ p.f.u. per mouse. To evaluate the induction of immune responses and the protective capacity of the recombinant viruses against wild-type HPAI H5N1 virus challenge, mice (20 per group) were immunized i.n. with recombinant viruses in a 50 μl volume at 10^6^ EID_50_ per mouse. All mock-immunized mice received 50 μl PBS. At 21 days post-inoculation (p.i.), sera were collected for antibody titration. At 21 days p.i., mice (10 per group) were challenged with 10^5^ p.f.u. (20 MLD_50_) WSN virus or 20 EID_50_ (20 MLD_50_) HPAI H5N1 virus by the intranasal route. Alternatively, mice (10 per group) received a booster immunization at 21 days post-vaccination, and 21 days later were challenged as described above. At 3 days post-challenge (p.c.) (and 6 days p.c. where indicated), three mice per group were sacrificed and their lungs collected and homogenized to measure virus titres. Lung homogenates were prepared in PBS and frozen at −70 °C until use. Virus titres in lung homogenates were determined by plaque assay (WSN) or as TCID_50_ (HPAI H5N1) on MDCK cells at 37 °C.

### Microneutralization assays.

Sera treated with receptor destroying enzyme were serially diluted twofold in PBS and placed into 96-well U-bottomed microtitre plates (50 μl per well). Following the addition of 50 μl containing 100 TCID_50_ virus diluted in PBS into each well, the plates were mixed and incubated at 37 °C for 1 h. Subsequently, the serum/virus mixture (100 μl) was added to a monolayer of MDCK cells in a 96-well plate. The plate was incubated at 4 °C for 15 min and then transferred to 37 °C for 45 min. After incubation, the serum/virus mixture was removed and 200 μl Opti-MEM I with 1 μg TPCK-trypsin ml^−1^ was added. The cells were incubated at 37 °C for 3 days and an HA assay was performed. Neutralizing antibody titres were expressed as the reciprocal of the highest dilution of the sample that completely inhibited haemagglutination. HA assays were performed following the recommendations of WHO/OIE.

## RESULTS

### *In vitro* characterization of recombinant viruses carrying the internal genes of the genetically modified influenza A/Guinea fowl/Hong Kong/WF10/99 (H9N2) virus

The *ts* phenotype of the influenza A/Ann Arbor/6/60 (H2N2) MDV-A strain has been mapped to 3 aa mutations in PB1 (K391E, E581G and A661T), one in PB2 (N265S) and one in NP (D34G) (Jin *et al.*, 2004). We showed previously that the *ts* loci in the PB2 and PB1 genes of the MDV-A strain could be transferred to the WF10 virus backbone producing a similar *ts* phenotype (Song *et al.*, 2007) and that the addition of an HA tag at the C terminus of the PB1 gene provided an attenuated phenotype in chickens and quail. To characterize further the biological properties of attenuated viruses using the WF10 backbone and to determine their potential as universal vaccine donors, we created additional recombinant viruses and tested them *in vitro*. We rescued three recombinant viruses, called 6WF10 : 2H1N1, 6WF10*ts* : 2H1N1 and 6WF10*att* : 2H1N1. The 6WF10 : 2H1N1 virus contains the internal genes of the WF10 virus and the HA and NA genes of the influenza A/WSN/33 (H1N1) virus. The genetic background of the 6WF10*ts* : 2H1N1 virus was the same as the 6WF10 : 2H1N1 virus, except that the PB2 and PB1 genes carried the *ca*/*ts*/*att* MDV-A mutations. The 6WF10*att* : 2H1N1 virus carried the *ca*/*ts*/*att* loci and HA tag modification.

We analysed the growth characteristics of the recombinant viruses at different temperatures in MDCK cells. The recombinant viruses grew as efficiently as the wild-type virus in eggs incubated at 35 °C, with titres ≥7.0 log_10_ p.f.u. ml^−1^ (Table 1). Plaque formation in MDCK cells for the 6WF10*ts* : 2H1N1 and 6WF10*att* : 2H1N1 viruses was impaired at 37 °C compared with the 6WF10 : 2H1N1 virus, which is consistent with the presence of *ts* mutations in their respective backbones. The 6WF10*att* : 2H1N1 and 6WF10*ts* : 2H1N1 viruses produced relatively larger plaques and grew better at 32 °C than at 37 or 38.5 °C (Table 1). As expected, the 6WF10 : 2H1N1 virus did not show a significant reduction in plaque numbers at 37 °C and only a slight 0.5 log_10_ reduction at 38.5 °C. In contrast, plaque formation by the 6WF10*ts*: 2H1N1 virus was reduced by 0.6 log_10_ at 37 °C and 3.4 log_10_ at 38.5 °C, respectively, compared with at 32 °C. The 6WF10*att* : 2H1N1 double-mutant virus had plaque numbers that were reduced by 1.0 log_10_ at 37 °C and was unable to produce plaques at 38.5 °C, which is consistent with our previous observations. These studies suggested that the *ts* phenotype in our WF10 backbone would be manifested, regardless of the surface genes.

### Genetically modified WF10*att* viruses with H1N1 surface genes are attenuated in mice

**Fig. 1. f1:**
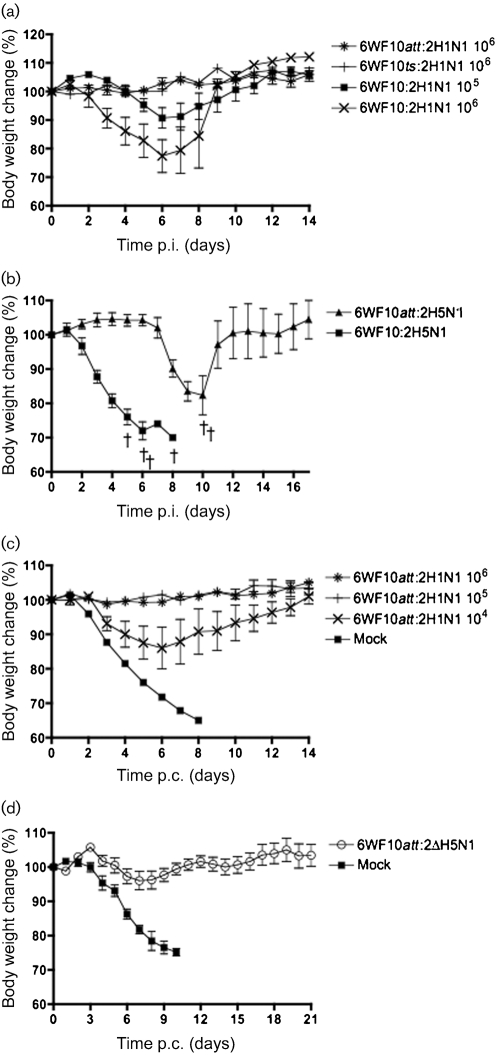
Attenuation and protective efficacy of the genetically modified WF10 influenza virus backbone in mice. (a) Mice (four per group) were infected with the indicated doses (p.f.u.) of recombinant H1N1 viruses. (b) Mice (four per group) were infected (10^6^ EID_50_ per mouse) with the indicated recombinant H5N1 viruses carrying an HPAI H5 HA protein. Days on which mice died are indicated by †. (c) Mice (four per group) were vaccinated with the indicated doses (p.f.u.) of the 6WF10*att* : 2H1N1 virus and 21 days later were challenged with 20 MLD_50_ per mouse of the WSN H1N1 virus. (d) Mice (four per group) were vaccinated with 10^6^ EID_50_ per mouse of the 6WF10*att* : 2ΔH5N1 virus and 21 days later challenged with 20 MLD_50_ per mouse of the HPAI H5N1 virus. In all experiments, changes in body weight and clinical signs of disease were followed over time.

Mice were inoculated with different doses of the WSN wild-type, 6WF10 : 2H1N1, 6WF10*ts* : 2H1N1 or 6WF10*att* : 2H1N1 virus (only doses for 10^5^ and 10^6^ p.f.u. for the WSN and 10^6^ p.f.u. for the mutant viruses are shown; Table 2). Severe clinical symptoms were observed in mice infected with WSN. Four of four mice died within 8 days when inoculated with 10^6^ or 10^5^ p.f.u. virus (Table 2). Similarly, mice infected with the 6WF10 : 2H1N1 virus showed severe signs of disease and half of them (two of four) died when inoculated with 10^6^ p.f.u. virus (Table 2). A noticeable reduction in body weight was also observed when mice were inoculated with 10^5^ p.f.u. 6WF10 : 2H1N1 virus (Fig. 1a[Fig f1]). In contrast, mice infected with the 6WF10*ts* : 2H1N1 or the 6WF10*att* : 2H1N1 virus exhibited no clinical signs of influenza infection and none of them died (Fig. 1a[Fig f1]). These results indicated that the 6WF10*ts* : 2H1N1 and 6WF10*att* : 2H1N1 viruses are attenuated in mice.

Next, we checked the replication of recombinant viruses in mouse lungs. As shown in Table 3, the 6WF10*ts* : 2H1N1 virus replicated poorly in mouse lungs. The growth of the 6WF10*ts* : 2H1N1 virus in mouse lungs was approximately 1.4 and 3.8 log_10_ lower than the 6WF10 : 2H1N1 or WSN virus. This difference was greater when mice were inoculated with a lower dose of virus. Furthermore, the double-mutant 6WF10*att* : 2H1N1 virus was even more attenuated in mice than the 6WF10*ts* : 2H1N1 virus (Table 3). Thus, the growth of the 6WF10*att* : 2H1N1 virus was highly restricted in mice and this was consistent with our *in vitro* plaque-reduction assays.

### Attenuation in mice of WF10*att* viruses in the context of H5N1 and H7N2 subtypes

The attenuated phenotype of WF10 recombinant viruses carrying the HA and NA genes of an HPAI H5N1 virus, its low-pathogenicity version with the polybasic region of the H5 HA removed (ΔH5N1) and H7N2 subtypes was evaluated in mice (Table 2). Mice inoculated with the 6WF10 : 2H5N1 strain, which resembles a wild-type HPAI H5N1 virus, showed severe clinical symptoms (Fig. 1b[Fig f1]) and four of four mice died within 8 days (Fig. 1b[Fig f1]). Interestingly, mice infected with the 6WF10*att* : 2H5N1 virus – carrying the WF10*att* virus backbone and the wild-type HPAI H5N1 surface genes – showed a less severe outcome of disease. Although two of four mice died, the WF10*att* was noticeably less virulent than the 6WF10 : 2H5N1 or the HPAI H5N1 wild-type virus. This observation was further confirmed by MLD_50_ assays, which required 10^6^ EID_50_ of the 6WF10*att *: 2H5N1 virus compared with <10^2^ EID_50_ for the 6WF10 : 2H5N1 virus (the exact lower limit was not tested) or 1 EID_50_ for the HPAI H5N1 virus (data not shown). These results highlight the attenuated nature of the WF10*att* backbone, even in the context of HPAI H5N1 surface genes. As expected, mice infected with the 6WF10*att* : 2ΔH5N1 or 6WF10*att* : 2H7N2 virus exhibited no clinical signs of influenza infection and none of them died (Table 2). The 6WF10*att* : 2ΔH5N1 virus was not detected in the lungs; however, the 6WF10*att* : 2H7N2 virus was detected in the lungs at 3 days p.i. (Table 3). The limited, although clearly discernible, replication of the 6WF10*att* : 2H7N2 virus in mouse lungs contrasted with the absence of the virus in chickens and quail lungs as described previously (Song *et al.*, 2007). It should be noted that the 6WF10*att* : 2H7N2 obtained from mouse lungs at 3 days p.i. was not a non-attenuated revertant strain. For reasons that are beyond the scope of this report, the 6WF10*att* : 2H7N2 virus showed better replication in mouse lungs than the 6WF10*att* : 2H1N1 or 6WF10*att* : 2ΔH5N1 virus (Table 3). Nevertheless, these results indicated that the WF10*att* backbone is attenuated in mice, whichever surface proteins are present.

### The WF10*att* backbone provides protection in mice against homologous challenge with lethal H1N1 or H5N1 subtype

In order to determine the protective efficacy of the 6WF10*att* backbone for mice against the WSN virus, we immunized mice i.n. with 10^4^, 10^5^ or 10^6^ p.f.u. of the 6WF10*att* : 2H1N1 virus. At 21 days p.i., mice were challenged with a lethal dose of the virulent WSN virus. Mice immunized with the 6WF10*att* : 2H1N1 virus survived the challenge with no signs of disease, although a significant decrease in body weight was observed in the group immunized with the lowest vaccine dose (Fig. 1c[Fig f1]). In contrast, the mock-immunized group developed severe pneumonia, showed drastic body weight loss and eventually died by 8 days p.i. (Fig. 1c[Fig f1]). More importantly, mice vaccinated with 10^6^ p.f.u. of the 6WF10*att* : 2H1N1 virus showed a significant reduction in the level of challenge virus isolated from lungs by 3 days p.i. in contrast to the mock-vaccinated mice (Table 4). These data suggested that, at doses that showed either very limited or undetectable replication in the mouse lung, the 6WF10*att* : 2H1N1 virus was able to induce immune responses that completely protected mice from challenge with the lethal WSN virus.

**Fig. 2. f2:**
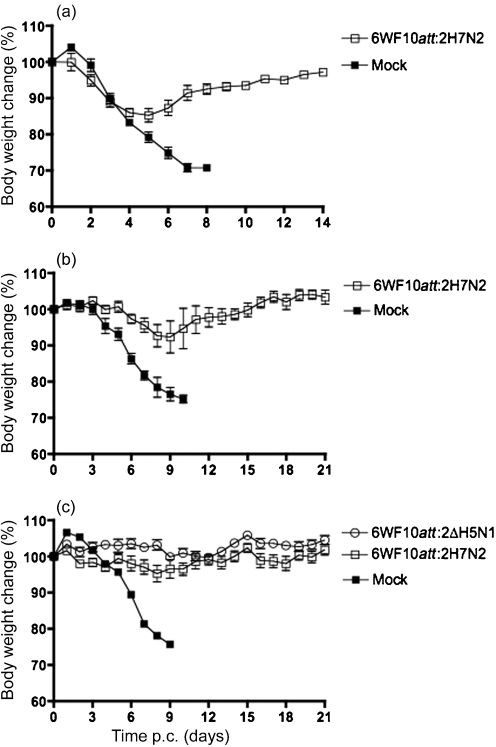
Heterologous protection in mice after single-dose and booster immunization regimes using an attenuated WF10 H7N2 virus. (a) Mice (four per group) were vaccinated with 10^6^ EID_50_ per mouse of the 6WF10*att* : 2H7N2 virus and challenged 21 days later with 20 MLD_50_ per mouse of the WSN H1N1 virus. (b) Mice (four per group) were vaccinated with 10^6^ EID_50_ per mouse of the 6WF10*att* : 2H7N2 virus and challenged 21 days later with 20 MLD_50_ per mouse of the HPAI H5N1 virus. (c) Mice (four per group) were vaccinated with 10^6^ EID_50_ per mouse of the 6WF10*att* : 2H7N2 virus. At 21 days p.i., mice received a second dose of the 6WF10*att* : 2H7N2 virus, and 21 days after this booster immunization, mice were challenged with 20 MLD_50_ per mouse of the HPAI H5N1 virus. In all experiments, changes in body weight and clinical signs of disease were followed over time.

We next evaluated the protection of the recombinant 6WF10*att* : 2ΔH5N1 against HPAI H5N1 challenge. As shown in Fig. 1(d), all mice vaccinated with 6WF10*att* : 2ΔH5N1 were protected against lethal HPAI H5N1 challenge. A slight body weight loss (about 10 %) was evident between 5 and 8 days p.c. All mice gained body weight thereafter without overt signs of disease. In contrast, mock-vaccinated mice died by day 10 (Fig. 1d[Fig f1]). Virus clearance was monitored at days 3 and 6 p.c. As shown in Table 4, virus titres within the lungs were significant, although a very slight reduction was observed in mice vaccinated with 6WF10*att* : 2ΔH5N1 at 6 days p.c. with a reduction of 0.6 log_10_ TCID_50_. As a significant amount of virus was detected in the immunized mice at 3 and 6 days p.c., despite complete protection against the HPAI H5N1 virus, we wanted to test whether a booster immunization would result in a better response and faster virus clearance (Table 4 and Fig. 2c[Fig f2]). Our results suggested that booster immunization improved the overall response to HPAI H5N1 challenge. No significant body weight loss was detected in the boosted mice, whilst a substantial reduction in challenged virus was seen in the booster group at 6 days p.c.: only 2.3 log_10_ TCID_50_ virus was present compared with single immunization where 4.9 log_10_ TCID_50_ virus was present. These results suggested that the single dose of the WF10*att* backbone can protect mice against homologous virus challenge, but that a booster leads to faster virus clearance.

### The WF10*att* backbone provides protection in mice against heterologous challenge

In order to understand whether intranasal immunization of recombinant viruses induced cross protective immunity against H1N1 or H5N1 viruses, groups of seven or 10 mice were immunized with a heterologous subtype, 6WF10*att* : 2H7N2 virus (Song *et al.*, 2007). Mice immunized with a single dose of 6WF10*att* : 2H7N2 survived the lethal challenge with both the WSN virus and HPAI H5N1 (Fig. 2a, b[Fig f2]). Mice immunized with the 6WF10*att* : 2H7N2 virus showed some body weight loss (about 15 % between days 4 and 6), although they all survived the challenge. These results suggested that the WF10*att* backbone is capable of providing cross-protective immunity against two different lethal virus challenges. Interestingly, significant virus titres were found at 3 and 6 days p.c. in the lungs of mice challenged with the HPAI H5N1 virus (Table 4). We investigated whether improved clearance of the challenged virus could be achieved following a booster vaccination regime (Table 4 and Fig. 2c[Fig f2]). Mice that received two doses of the 6WF10*att* : 2H7N2 virus showed minimal weight loss, displayed no disease signs and were completely protected from challenge with HPAI H5N1. However, the single-dose and booster immunization groups had similar levels of challenge HPAI H5N1 virus at 3 or 6 days p.c. (Table 4). It should be noted that heterologous protection was not necessarily due to the ability of the 6WF10*att* : 2H7N2 virus to replicate in mouse lungs. Using another WF10*att* subtype virus, the 6WF10*att* : 2H9N2 virus, which did not replicate in mouse lungs, we achieved similar levels of cross-protection (data not shown). These results suggest that protection by the 6WF10*att* : 2H7N2 virus is probably provided by cell-mediated mechanisms that do not prevent initial replication of the HPAI H5N1 virus. Thus, the WF10*att* backbone provides protection in mice against heterologous challenge with either WSN or HPAI H5N1 virus, although it does not prevent virus replication at the early stages of infection.

### Significant variations in the ability of recombinant WF10*att* viruses to induce neutralizing antibody responses

To evaluate the immune responses induced by the WF10*att* viruses that protected mice against lethal challenge with WSN and HPAI H5N1, we determined the levels of neutralizing antibody in the sera of immunized mice using microneutralization assays. As shown in Table 5, discernible and adequate neutralizing responses were observed in mice immunized with the 6WF10*att* : 2H1N1 virus that were similar to those obtained using either the 6WF10 : 2H1N1 or WSN virus (data not shown). Lower neutralizing antibody titres were observed in the pooled sera of the four surviving mice immunized with a single dose of 6WF10*att* : 2H7N2 virus against its homologous virus; however, after the booster, an increased neutralizing antibody titre was clearly observed (Table 5). As expected, the 6WF10*att* : 2H7N2 virus showed no cross-reactive antibodies that could neutralize the heterologous WSN or H5N1 virus. Interestingly, mice vaccinated with the 6WF10*att* : 2ΔH5N1 virus showed no discernible neutralizing antibody reaction, even after booster immunization. These data suggested that survival of mice from challenge is not solely dependent on neutralizing antibodies; rather, there may be a combination of humoral and cell-mediated responses.

## DISCUSSION

Recent studies have indicated that transferring the *ts* amino acid signature of the MDV-A virus into different human influenza strains results in a *ts* phenotype *in vitro* and attenuation in ferrets (Jin *et al.*, 2004). Because of the transferable nature of the *ts* mutations of the MDV-A virus, we sought to determine whether such mutations would impart a similar phenotype to an AIV. For this purpose, we chose a virus that has demonstrated a broad host range in order to generate an attenuated virus backbone that could be used for the development of a universal vaccine for multiple animal species, i.e. from poultry to humans. We chose the internal genes of the AIV A/Guinea fowl/Hong Kong/WF10/99 (H9N2), which replicates and transmits efficiently in birds, causes respiratory disease in mice without adaptation and replicates efficiently in ferrets (H. Wan and others, unpublished data) (Choi *et al.*, 2004). We successfully generated attenuated H1N1, H5N1 and H7N2 reassortant viruses with the internal genes from the WF10*att* virus backbone.

There are obvious limitations in the preparation of influenza vaccine stocks in the advent of a pandemic that are inherent to the rapid mutability of the virus. Thus, it is not possible to predict whether the antigenic make-up of the vaccine seed stock would confer protective immunity against the pandemic strain. Meanwhile, the world is experiencing a pandemic of influenza in birds caused by an H5N1 virus in which multiple domestic and wild avian species are involved (Webster *et al.*, 2007). Although this H5N1 virus has been restricted to Eurasia and some countries in Africa, there is a latent risk that this virus may spread worldwide. The H5N1 virus has also shown an unusually expanded host range, i.e. not only have birds and humans been infected and died, but feline species, otherwise regarded as resistant to influenza, have also experienced a similar fate. In fact, little is known about the extent of the host range of the H5N1 virus in nature. Culling and quarantine complemented with the use of vaccines are being implemented to control the spread of the H5N1 virus in domestic poultry and to minimize the risk of human exposure (Capua & Alexander, 2002, 2004; Capua & Marangon, 2004). Approved vaccines for poultry rely on inactivated vaccines or a fowlpox recombinant virus (Capua *et al.*, 2003). Parenteral administration of these vaccines limits their use in mass vaccination campaigns. The magnitude of an H5N1 outbreak may be managed or prevented with vaccination strategies performed by aspersion, *in ovo* or by drinking water, so that thousands of birds can be immunized at the same time with few labour costs. A second issue has recently emerged during the preparation of inactivated vaccines and is related to the human health risks of personnel exposed to AIVs whose interspecies potential is poorly defined.

Previously, we showed the WF10*att* backbone was able to replicate in the upper respiratory tracts of chickens and no or very little virus was found in the lungs or cloaca, suggesting attenuation in chickens (Song *et al.*, 2007). In this paper, we have shown that incorporation of the *ts* loci of MDV-A and an HA tag in the PB1 gene of WF10 resulted in a virus that was highly attenuated in mice. Mice infected with 10^6^ p.f.u. 6WF10*att* : 2H1N1 (Fig. 1a[Fig f1], Tables 2 and 3), 10^6^ EID_50_ 6WF10*att*:2ΔH5N1 (Tables 2 and 3) or 6WF10*att* : 2H7N2 (Tables 2 and 3) showed no clinical signs of disease, very little virus was detected 3 days p.i. in mouse lungs and none of the mice died, all indicating that the WF10*att* backbone is attenuated in mice, whichever surface genes are present.

As the recombinant WF10*att* viruses were attenuated in mice, we determined the protective efficacy of these viruses. Mice immunized with a single dose (10^6^ p.f.u.) of 6WF10*att* : 2H1N1 or 6WF10*att* : 2H7N2 survived challenge with 20 MLD_50_ of lethal WSN, and the 6WF10*att* : 2H1N1-vaccinated mice were able to clear the challenge virus from their lungs by 3 days p.c. In the case of HPAI H5N1 challenge, mice immunized with a single dose (10^6^ EID_50_) or given a booster 21 days after a single dose of 6WF10*att* : 2ΔH5N1 or 6WF10*att* : 2H7N2 survived challenge. Unlike the results with the lethal WSN challenge at 3 and 6 days p.c., virus remained in the lungs p.c. although the mice were completely protected. These data suggested that, at doses that showed either very limited or undetectable replication in the mouse lung, the 6WF10*att* recombinant viruses were able to induce immune responses that completely protected mice from challenge with the lethal WSN or HPAI H5N1 virus. Using microneutralization assays, we determined the neutralizing antibody titres induced by the WF10*att* viruses that protected the vaccinated mice against lethal WSN and HPAI H5N1 challenge. Adequate neutralizing antibodies were observed in mice immunized with 6WF10*att* : 2H1N1, but no neutralizing antibodies were observed in mice immunized with a single dose or booster of 6WF10*att* : 2ΔH5N1, although mice were completely protected. These results are consistent with previous observations where neutralizing antibody titres against the HPAI H5N1 virus were undetectable even after booster immunization, despite 100 % survival in challenge studies (Lu *et al.*, 2006). Although 6WF10*att* : 2H7N2-immunized mice generated a lower titre of neutralizing antibodies after the first dose, there was an increase in neutralizing antibody titre after the booster dose. We did not observe cross-reactive neutralizing antibodies to the challenge viruses, WSN or A/Vietnam/1203/04 (H5N1). Although it remains unclear how WF10*att* protects, together these observations strongly suggest that cell-mediated responses are involved in protecting mice immunized with WF10*att* viruses against lethal challenge with WSN or HPAI H5N1 virus.

For a pandemic, and from a practical point of view, it would be ideal to prepare vaccine seed stocks that can be used in multiple animal species. We explored this latter possibility and generated an attenuated AIV with an extended host range that could be used for the preparation of vaccines for either birds or mammals. The use of a universal backbone obviates the need for the reformulation of a vaccine specifically designed for use in humans, which would save valuable time as the vaccine itself could already be in use for other animal species. A live attenuated AIV vaccine for poultry would be amenable to mass vaccination and would negate the limitations associated with recombinant approaches in terms of prior exposure to the wild-type virus. The potential of reassortment of the surface genes of our vaccine virus with a wild-type virus would limit its use in domestic birds, although this risk could be greatly minimized by performing *in ovo* vaccination, as we have shown recently (Song *et al.*, 2007). Our approach should also allow the mass vaccination of wild bird species in which the H5N1 virus appears to have gone through cycles of increased virulence, the ecological consequences of which remain to be seen. Our approach also has the potential for vaccination of domestic pets, such as cats and dogs, which have also been involved in recent H5N1 influenza outbreaks (Amonsin *et al.*, 2007; Songserm *et al.*, 2006a, b). Thus, our strategy provides an alternative approach for the preparation of vaccines for epidemic and pandemic influenza.

## Figures and Tables

**Table 1. t1:** Reduction in recombinant virus titres at the indicated temperatures compared with the permissive temperature (32 °C) np, No plaques detected.

**Virus**	**Plaque reduction (log_10_ p.f.u. ml^−1^)**		Stock virus titre (log_10_ p.f.u. ml^−1^)*
	**37 °C**	**38.5 °C**	
6WF10*att* : 2H1N1	1.0	np	7.3
6WF10*ts* : 2H1N1	0.6	3.4	7.6
6WF10 : 2H1N1	−0.1	0.5	7.4
WSN	0.3	1.2	7.3

*Viruses were grown for 48 h in the allantoic cavity of 10-day-old embryonated chicken eggs. The amount of virus in the allantoic fluid was determined by plaque assay on MDCK cells at 32 °C. Results represent the mean of two independent experiments.

**Table 2. t2:** Survival of mice following infection with recombinant viruses generated by reverse genetics

**Virus**	**Infection dose**	Survival (no. survivors/no. tested)*
A/WSN/33 (H1N1)	10^6^†	0/4
	10^5^†	0/4
6WF10 : 2H1N1	10^6^†	2/4
6WF10*att* : 2H1N1	10^6^†	4/4
A/Vietnam/1203/04 (H5N1)	10^6^‡	0/4
6WF10 : 2H5N1	10^6^‡	0/4
6WF10*att* : 2H5N1	10^6^‡	2/4
6WF10*att* : 2ΔH5N1	10^6^‡	4/4
6WF10*att* : 2H7N2	10^6^‡	4/4

*Survival of mice was monitored for 14 days p.i. †Titre in p.f.u. ‡Titre in EID_50_.

**Table 3. t3:** Replication of recombinant vaccine viruses in mouse lungs at 3 days post-infection Mice were inoculated i.n. with virus at the indicated dose. At 3 days post-infection, lungs were collected and homogenized for virus titration. Data are the means of virus titres from four mice in each group. −, Not tested; nd, not detected; bld, below limit of detection.

**Virus**	**Infectious virus dose used**			
	**10^6^**	**10^5^**	**10^4^**	**10^3^**
WSN	−	7.1±0.1*	6.8±0.1*	5.5±0.2*
6WF10 : 2H1N1	−	4.7±0.1*	4.2±0.3*	3.0±0.2*
6WF10*ts* : 2H1N1	−	3.3±0.3*	2.2±0.2*	bld
6WF10*att* : 2H1N1	bld	bld	−	−
6WF10*att* : 2ΔH5N1	bld	−	−	−
6WF10*att* : 2H7N2	2.9±0.8†	−	−	−

*Titre in log_10_ p.f.u. per lung. †Titre in log_10_ EID_50_ per lung.

**Table 4. t4:** Clearance of challenge virus in mice immunized with recombinant vaccine virus

**Immunized with**	**Immunization dose (p.f.u. or EID_50_) per mouse)**	Challenged with 20 MLD_50_ of:*	**Challenge virus titre at 3 days p.c. (log_10_ p.f.u./TCID_50_ per lung)**	**Challenge virus titre at 6 days p.c. (log_10_ TCID_50_ per lung)**
PBS†	–	WSN	7.3±0.1	−
WSN†	10^3^	WSN	bld	−
6WF10*att* : 2H1N1†	10^6^	WSN	bld	−
6WF10*att* : 2H1N1†	10^5^	WSN	2.8±0.9	−
PBS‡		HPAI H5N1	5.7±0.3	7.1±0.3
6WF10*att* : 2ΔH5N1‡	10^6^	HPAI H5N1	5.5±0.4	4.9±0.1
6WF10*att* : 2ΔH5N1+booster‡	10^6^	HPAI H5N1	6.8±0.2	2.3±0.1
6WF10*att* : 2H7N2‡	10^6^	HPAI H5N1	5.0±0.3	5.1±0.3
6WF10*att* : 2H7N2+booster	10^6^	HPAI H5N1	5.8±0.2	5.8±0.8

*Immunized mice were challenged with 10^5^ p.f.u. WSN virus or 20 EID_50_ HPAI H5N1 (equivalent to 20 MLD_50_). At 3 or 6 days post-immunization, the lungs were collected and homogenized, and virus titres were assayed by plaque assay or TCID_50_ determination in MDCK cells. The data indicate the mean lung virus titre±sd from three mice per group. bld, Below limit of detection; −, not tested. †Results given in p.f.u. ‡Results given as TCID_50_.

**Table 5. t5:** Microneutralization (MN) antibody titres in mouse sera against homologous and heterologous viruses

**Immunized with:**	**Immunization dose**	MN titres against homologous virus*	**MN titres against WSN**	**MN titres against H5N1**
PBS		<10	<10	<10
6WF10*att* : 2H1N1	10^6^†	160	160	<10
6WF10*att* : 2H1N1	10^5^†	80	80	<10
6WF10*att* : 2ΔH5N1	10^6^‡	<10	<10	<10
6WF10*att* : 2ΔH5N1+booster	10^6^‡	<10	<10	<10
6WF10*att* : 2H7N2	10^6^‡	40	<10	<10
6WF10*att* : 2H7N2+booster	10^6^‡	160	<10	<10

*Sera were collected at 21 days post-immunization. The data represent pooled sera from four mice per group. MN assays were performed using homologous viruses: A/WSN/33 (H1N1), A/Vietnam/1203/04 (ΔH5N1) and A/Chicken/Delaware/VIVA/04 (H7N2). †Titre in p.f.u. ‡Titre in TCID_50_.

## References

[r1] Amonsin, A., Songserm, T., Chutinimitkul, S., Jam-On, R., Sae-Heng, N., Pariyothorn, N., Payungporn, S., Theamboonlers, A. & Poovorawan, Y. (2007). Genetic analysis of influenza A virus (H5N1) derived from domestic cat and dog in Thailand. Arch Virol 152, 1925–1933. 17577611 10.1007/s00705-007-1010-5

[r2] Belshe, R. B. (2004). Current status of live attenuated influenza virus vaccine in the US. Virus Res 103, 177–185. 15163507 10.1016/j.virusres.2004.02.031

[r3] Capua, I. & Alexander, D. J. (2002). Avian influenza and human health. Acta Trop 83, 1–6. 12062786 10.1016/s0001-706x(02)00050-5

[r4] Capua, I. & Alexander, D. J. (2004). Avian influenza: recent developments. Avian Pathol 33, 393–404. 15370036 10.1080/03079450410001724085

[r5] Capua, I. & Marangon, S. (2004). Vaccination for avian influenza in Asia. Vaccine 22, 4137–4138. 15474703 10.1016/j.vaccine.2004.04.017

[r6] Capua, I., Terregino, C., Cattoli, G., Mutinelli, F. & Rodriguez, J. F. (2003). Development of a DIVA (Differentiating Infected from Vaccinated Animals) strategy using a vaccine containing a heterologous neuraminidase for the control of avian influenza. Avian Pathol 32, 47–55. 12745380 10.1080/0307945021000070714

[r7] Choi, Y. K., Ozaki, H., Webby, R. J., Webster, R. G., Peiris, J. S., Poon, L., Butt, C., Leung, Y. H. & Guan, Y. (2004). Continuing evolution of H9N2 influenza viruses in Southeastern China. J Virol 78, 8609–8614. 15280470 10.1128/JVI.78.16.8609-8614.2004 PMC479067

[r8] Guan, Y., Shortridge, K. F., Krauss, S., Chin, P. S., Dyrting, K. C., Ellis, T. M., Webster, R. G. & Peiris, M. (2000). H9N2 influenza viruses possessing H5N1-like internal genomes continue to circulate in poultry in southeastern China. J Virol 74, 9372–9380. 11000205 10.1128/jvi.74.20.9372-9380.2000 PMC112365

[r9] Harper, S. A., Fukuda, K., Uyeki, T. M., Cox, N. J. & Bridges, C. B. (2004). Prevention and control of influenza: recommendations of the Advisory Committee on Immunization Practices (ACIP). MMWR Recomm Rep 53, 1–40. 15163927

[r10] Hoffmann, E., Neumann, G., Kawaoka, Y., Hobom, G. & Webster, R. G. (2000). A DNA transfection system for generation of influenza A virus from eight plasmids. Proc Natl Acad Sci U S A 97, 6108–6113. 10801978 10.1073/pnas.100133697 PMC18566

[r11] Horimoto, T. & Kawaoka, Y. (2001). Pandemic threat posed by avian influenza A viruses. Clin Microbiol Rev 14, 129–149. 11148006 10.1128/CMR.14.1.129-149.2001 PMC88966

[r12] Jin, H., Zhou, H., Lu, B. & Kemble, G. (2004). Imparting temperature sensitivity and attenuation in ferrets to A/Puerto Rico/8/34 influenza virus by transferring the genetic signature for temperature sensitivity from cold-adapted A/Ann Arbor/6/60. J Virol 78, 995–998. 14694130 10.1128/JVI.78.2.995-998.2004 PMC368857

[r13] Lin, Y. P., Shaw, M., Gregory, V., Cameron, K., Lim, W., Klimov, A., Subbarao, K., Guan, Y., Krauss, S. & other authors (2000). Avian-to-human transmission of H9N2 subtype influenza A viruses: relationship between H9N2 and H5N1 human isolates. Proc Natl Acad Sci U S A 97, 9654–9658. 10920197 10.1073/pnas.160270697 PMC16920

[r14] Lu, X., Edwards, L. E., Desheva, J. A., Nguyen, D. C., Rekstin, A., Stephenson, I., Szretter, K., Cox, N. J., Rudenko, L. G. & other authors (2006). Cross-protective immunity in mice induced by live-attenuated or inactivated vaccines against highly pathogenic influenza A (H5N1) viruses. Vaccine 24, 6588–6593. 17030078 10.1016/j.vaccine.2006.05.039

[r15] Maassab, H. F. (1967). Adaptation and growth characteristics of influenza virus at 25 °C. Nature 213, 612–614. 6040602 10.1038/213612a0

[r16] Murphy, B. R., Sly, D. L., Tierney, E. L., Hosier, N. T., Massicot, J. G., London, W. T., Chanock, R. M., Webster, R. G. & Hinshaw, V. S. (1982). Reassortant virus derived from avian and human influenza A viruses is attenuated and immunogenic in monkeys. Science 218, 1330–1332. 6183749 10.1126/science.6183749

[r17] Murphy, B. R., Park, E. J., Gottlieb, P. & Subbarao, K. (1997). An influenza A live attenuated reassortant virus possessing three temperature-sensitive mutations in the PB2 polymerase gene rapidly loses temperature sensitivity following replication in hamsters. Vaccine 15, 1372–1378. 9302747 10.1016/s0264-410x(97)00031-5

[r18] Neumann, G. & Kawaoka, Y. (2001). Reverse genetics of influenza virus. Virology 287, 243–250. 11531402 10.1006/viro.2001.1008

[r19] Peiris, M., Yuen, K. Y., Leung, C. W., Chan, K. H., Ip, P. L., Lai, R. W., Orr, W. K. & Shortridge, K. F. (1999). Human infection with influenza H9N2. Lancet 354, 916–917. 10489954 10.1016/s0140-6736(99)03311-5

[r20] Peiris, J. S., Guan, Y., Markwell, D., Ghose, P., Webster, R. G. & Shortridge, K. F. (2001). Cocirculation of avian H9N2 and contemporary “human” H3N2 influenza A viruses in pigs in southeastern China: potential for genetic reassortment? J Virol 75, 9679–9686. 11559800 10.1128/JVI.75.20.9679-9686.2001 PMC114539

[r21] Perez, D. R., Lim, W., Seiler, J. P., Yi, G., Peiris, M., Shortridge, K. F. & Webster, R. G. (2003a). Role of quail in the interspecies transmission of H9 influenza A viruses: molecular changes on HA that correspond to adaptation from ducks to chickens. J Virol 77, 3148–3156. 12584339 10.1128/JVI.77.5.3148-3156.2003 PMC149770

[r22] Perez, D. R., Webby, R. J., Hoffmann, E. & Webster, R. G. (2003b). Land-based birds as potential disseminators of avian mammalian reassortant influenza A viruses. Avian Dis 47, 1114–1117. 14575124 10.1637/0005-2086-47.s3.1114

[r23] Reed, L. J. & Muench, H. (1938). A simple method for estimating 50 percent endpoints. Am J Hyg 37, 493

[r24] Sears, S. D., Clements, M. L., Betts, R. F., Maassab, H. F., Murphy, B. R. & Snyder, M. H. (1988). Comparison of live, attenuated H1N1 and H3N2 cold-adapted and avian–human influenza A reassortant viruses and inactivated virus vaccine in adults. J Infect Dis 158, 1209–1219. 3198936 10.1093/infdis/158.6.1209

[r25] Song, H., Nieto, G. R. & Perez, D. R. (2007). A new generation of modified live-attenuated avian influenza viruses using a two-strategy combination as potential vaccine candidates. J Virol 81, 9238–9248. 17596317 10.1128/JVI.00893-07 PMC1951405

[r26] Songserm, T., Amonsin, A., Jam-on, R., Sae-Heng, N., Pariyothorn, N., Payungporn, S., Theamboonlers, A., Chutinimitkul, S., Thanawongnuwech, R. & Poovorawan, Y. (2006a). Fatal avian influenza A H5N1 in a dog. Emerg Infect Dis 12, 1744–1747. 17283627 10.3201/eid1211.060542 PMC3372347

[r27] Songserm, T., Amonsin, A., Jam-on, R., Sae-Heng, N., Meemak, N., Pariyothorn, N., Payungporn, S., Theamboonlers, A. & Poovorawan, Y. (2006b). Avian influenza H5N1 in naturally infected domestic cat. Emerg Infect Dis 12, 681–683. 16704821 10.3201/eid1204.051396 PMC3294706

[r28] Steinhoff, M. C., Halsey, N. A., Wilson, M. H., Burns, B. A., Samorodin, R. K., Fries, L. F., Murphy, B. R. & Clements, M. L. (1990). Comparison of live attenuated cold-adapted and avian–human influenza A/Bethesda/85 (H3N2) reassortant virus vaccines in infants and children. J Infect Dis 162, 394–401. 2197335 10.1093/infdis/162.2.394

[r29] Steinhoff, M. C., Halsey, N. A., Fries, L. F., Wilson, M. H., King, J., Burns, B. A., Samorodin, R. K., Perkis, V., Murphy, B. R. & Clements, M. L. (1991). The A/Mallard/6750/78 avian–human, but not the A/Ann Arbor/6/60 cold-adapted, influenza A/Kawasaki/86 (H1N1) reassortant virus vaccine retains partial virulence for infants and children. J Infect Dis 163, 1023–1028. 2019751 10.1093/infdis/163.5.1023

[r30] Subbarao, K., Webster, R. G., Kawaoka, Y. & Murphy, B. R. (1995). Are there alternative avian influenza viruses for generation of stable attenuated avian-human influenza A reassortant viruses? Virus Res 39, 105–118. 8837878 10.1016/0168-1702(95)00082-8

[r31] Wan, H. & Perez, D. R. (2007). Amino acid 226 in the hemagglutinin of H9N2 influenza viruses determines cell tropism and replication in human airway epithelial cells. J Virol 81, 5181–5191. 17344280 10.1128/JVI.02827-06 PMC1900221

[r32] Webster, R. G., Hulse-Post, D. J., Sturm-Ramirez, K. M., Guan, Y., Peiris, M., Smith, G. & Chen, H. (2007). Changing epidemiology and ecology of highly pathogenic avian H5N1 influenza viruses. Avian Dis 51, 269–272. 17494564 10.1637/7641-050206R.1

[r33] Xu, C., Fan, W., Wei, R. & Zhao, H. (2004). Isolation and identification of swine influenza recombinant A/Swine/Shandong/1/2003(H9N2) virus. Microbes Infect 6, 919–925. 15310468 10.1016/j.micinf.2004.04.015

[r34] Zangwill, K. M. & Belshe, R. B. (2004). Safety and efficacy of trivalent inactivated influenza vaccine in young children: a summary for the new era of routine vaccination. Pediatr Infect Dis J 23, 189–197. 15014289 10.1097/01.inf.0000116292.46143.d6

